# Who does not want to quit? Determinants of no intention to stop smoking in a population-based sample of current smokers

**DOI:** 10.1007/s11739-025-04174-0

**Published:** 2025-11-04

**Authors:** Yusuff Adebayo Adebisi, Najim Z. Alshahrani, Oshibe Joseph Daberechi, Yasir Ahmed Mohammed Elhadi, Don Lucero-Prisno Eliseo

**Affiliations:** 1 College of Social Sciences, University of Glasgow, 40 Bute Gardens, Glasgow, G12 8RT UK; 2 Department of Family and Community Medicine, Faculty of Medicine, University of Jeddah, Jeddah, Saudi Arabia; 3 Medical Laboratory Science Department, Ebonyi State University, Abakaliki, Nigeria; 4 College of Medicine and Health Sciences, Public Health Institute, United Arab Emirates University, 15551 Al Ain, United Arab Emirates; 5 Department of Global Health and Development, London School of Hygiene and Tropical Medicine, London, UK; 6 Center for University Research, University of Makati, Makati City, Philippines; 7 Research Office, Palompon Institute of Technology, Palompon, Leyte Philippines

**Keywords:** Tobacco smoking, Intention to quit, Smoking cessation, Current smokers, Scotland

## Abstract

Understanding why some people who smoke have no intention to quit is essential for designing inclusive tobacco control strategies that reach beyond already motivated individuals. This study aimed to identify factors associated with no intention to quit smoking among people who smoke in Scotland, analysing data from 2651 current smokers aged 16 years and older using the 2017, 2018, 2019, and 2021 waves of the Scottish Health Survey. The primary outcome was no intention to quit smoking. Multivariable logistic regression was used to assess sociodemographic, behavioural, and health-related predictors, applying survey weights to account for the complex sampling design. About one-third (34.1%) of those who currently smoke reported no intention to quit. Older adults (65 +) had significantly higher odds of no quit intention compared with those aged 16–24 years (adjusted odds ratio [aOR] = 2.74, 95% confidence interval [CI] 1.54–4.90, *p* = 0.001), as did those who began smoking before age 16 (aOR = 1.29, 95% CI 1.02–1.64, *p* = 0.031), had not received a doctor’s advice to quit (aOR = 1.38, 95% CI 1.09–1.74, *p* = 0.008), or did not know their daily cigarette consumption (aOR = 2.21, 95% CI 1.19–4.10, *p* = 0.012). In contrast, people with one or two prior quit attempts (aOR = 0.22, 95% CI 0.17–0.29, *p *< 0.001) or three or more attempts (aOR = 0.15, 95% CI 0.11–0.20, *p* < 0.001) had substantially lower odds of reporting no intention to quit compared with those who had never tried to quit. Similarly, current e-cigarette users (aOR = 0.57, 95% CI 0.40–0.81, *p* = 0.002) had lower odds of reporting no intention to quit than never users. No significant association was observed with socioeconomic deprivation, suggesting that while disadvantage shapes smoking prevalence, it may not directly determine quit intention. Reaching smokers with no quit intention may call for interventions that operate outside traditional clinical settings, including targeted outreach to older adults, early initiators, and those disconnected from healthcare services.

## Introduction

Cigarette smoking remains a significant global public health concern, accounting for over 8 million deaths annually, with more than 7 million of those deaths resulting from direct tobacco use and approximately 1.3 million due to non-smokers being exposed to secondhand smoke [1]. In the UK, smoking is the leading cause of preventable illness and early death, linked to cancers, cardiovascular disease, respiratory illness, and a wide range of comorbidities [2]. Despite substantial policy progress, including smoke-free legislation, tobacco taxation, mass media campaigns, and cessation services, tobacco use continues to impose a disproportionate burden on disadvantaged populations [3]. In Scotland, the smoking rate among adults has declined over the past two decades; however, as of the most recent reports, approximately 12–14% of Scottish adults still smoke, with rates nearly double among those in the most deprived areas compared to the least [4]. These disparities underline the complexity of socioeconomic inequality and smoking behaviours, highlighting the need to better understand subgroups of smokers who are not engaging with cessation support or motivated to quit.

The intention to quit smoking is a well-established predictor of future quit attempts and successful cessation [5, 6]. Behavioural models such as the Transtheoretical Model (Stages of Change) and the Theory of Planned Behaviour identify intention as a precursor to behavioural change [7]. Public health interventions often operate on the assumption that most smokers desire to quit, with efforts focused on providing tools and resources to support that intention. However, a growing body of research indicates that a notable proportion of smokers report no intention to stop smoking, even in the face of well-documented health risks [8, 9]. For instance, a US study estimated that 14% of smokers “never plan” to quit, and these individuals were more likely to deny the harms of smoking, underplay addiction, and report low worry about health consequences [8]. Cross-country evidence also suggests differences in quit motivation: Reid et al. found that smokers in the UK and US were less likely to intend to quit compared with their counterparts in Canada and Australia, highlighting the influence of national policy environments and cultural norms [5].

Smokers who report no intention to quit tend to disengage from cessation services, are underrepresented in quit-related studies, and may be overlooked by public health initiatives that predominantly target those already motivated to stop [5]. Their continued smoking not only worsens individual health outcomes, but also poses challenges for achieving population-level goals, including Scotland’s ambition to become tobacco free by 2034. While international evidence has identified sociodemographic, behavioural, and psychological factors associated with quit resistance [5, 6, 8, 9], it remains unclear how these patterns apply in Scotland, where smoking prevalence is persistently higher in disadvantaged groups. Addressing this gap is essential for developing interventions that go beyond “ready-to-quit” smokers and account for the deeper structural and psychological barriers that sustain tobacco use. To do so, this study examines the factors associated with no intention to quit smoking among smokers in Scotland and reports adjusted predictive probabilities by age and e-cigarette use to illustrate how quit resistance varies across subgroups.

## Methods

### Data source and study design

This study used data from the 2017, 2018, 2019, and 2021 waves of the Scottish Health Survey (SHeS), a nationally representative, cross-sectional survey of individuals living in private households across Scotland. The combined dataset included a total of 25,128 respondents: 5300 in 2017, 6790 in 2018, 6881 in 2019, and 6157 in 2021. The SHeS is designed to monitor the health and health-related behaviours of the Scottish population and to inform public health policy [10]. It employs a multistage stratified probability sampling method based on the postcode address file, ensuring representative coverage across geographical areas and demographic groups. Each annual wave is independently sampled, making the SHeS a repeated cross-sectional survey. The 2020 wave was excluded from this analysis due to disruptions in data collection and methodological changes during the COVID-19 pandemic, which rendered it non-comparable with other years [4]. Data collection was conducted via interviewer-led, computer-assisted personal interviews (CAPI), with sensitive questions completed by participants themselves to ensure greater privacy and accuracy [10].

### Study population

The combined dataset across the four survey waves included 25,128 respondents. After excluding 7161 individuals younger than 16 years and 15,316 non-smokers or inapplicable cases, the final analytic sample comprised 2651 respondents aged 16 years and older who were identified as current cigarette smokers.

### Outcome variable

The primary outcome was intention to quit smoking, based on the SHeS variable StopWant, labelled “Whether wants to give up smoking (CAPI)”. Current smokers were asked whether they wanted to give up smoking, with two possible responses: “Yes” and “No.” For analysis, responses were recoded into a binary variable: wants to quit (0) and does not want to quit (1).

### Predictor variables

Sociodemographic, behavioural, and health-related variables were selected based on previous literature and theoretical relevance [11–14]. Age was categorised into six groups: 16–24, 25–34, 35–44, 45–54, 55–64, and 65 years and above. Sex was coded as male or female. Socioeconomic status was assessed using the Scottish Index of Multiple Deprivation (SIMD), divided into quintiles ranging from most to least deprived. Marital status was grouped into married or cohabiting, single, and previously married. Educational attainment was classified into higher education (degree or equivalent), secondary or college education, and no formal education. Smoking behaviour variables included number of quit attempts (never tried, once or twice, or three times or more), and smoking intensity (light: < 10 cigarettes/day; moderate: 10–19/day; heavy: 20 +/day; or do not know amount smoked) E-cigarette use was categorised as current, former, or never use. Household smoking exposure was assessed by whether anyone smoked inside the home (yes or no). Alcohol consumption in the past 12 months was classified as frequent, occasional, or non-drinker. Age of smoking initiation was grouped into those who started before age 16 and those who started at age 16 or older. Self-rated general health was reported as very good/good, fair, or bad/very bad. Additional variables included whether a doctor had ever advised the respondent to quit smoking (yes or no), and whether the respondent reported having a long-standing illness (yes or no).

### Statistical analysis

Descriptive statistics were used to characterise the sample, comparing respondents with and without an intention to quit smoking. Differences across groups were assessed using Pearson’s Chi-square tests. Univariate (crude) logistic regression models were then fitted to estimate odds ratios (ORs) and 95% confidence intervals (CIs) for each predictor variable with the outcome. To identify independent associations, a multivariable logistic regression model was fitted including all covariates simultaneously. The final model was built based on theoretical importance and variables of interest, regardless of crude significance. Survey weights, primary sampling units (PSUs), and strata provided by SHeS were applied using the svyset and svy: commands in Stata to account for the complex survey design and ensure population-level estimates in the multivariable model.

Model diagnostics included checks for multicollinearity among the predictors using variance inflation factors (VIFs). A survey-adjusted goodness-of-fit test (Archer–Lemeshow F-adjusted mean residual test) was conducted to evaluate overall model fit, and the Brier score was calculated to assess predictive accuracy. In addition, predictive probabilities of having no intention to quit were estimated using the margins and marginsplot commands in Stata, adjusted for all predictors in the multivariable model, to provide subgroup predictions by age group and e-cigarette use.

As a sensitivity analysis, we additionally included survey year as a covariate in the regression models to assess possible year-to-year variation in the pooled 2017–2021 dataset. All analyses were conducted using Stata version 18, and significance was set at *p* < 0.05.

## Results

Of the 2651 current smokers in the analytic sample, 1747 (65.9%) expressed an intention to quit, while 904 (34.1%) reported no intention to stop smoking.

Table 1 presents the distribution of characteristics among current smokers by their intention to quit smoking. Intention to quit varied significantly across age groups (*χ*^2^ = 98.55, *p* < 0.001), with smokers aged 65 and above more likely to report no intention to quit (27.9%) compared with younger adults aged 16–24 (7.1%) or 25–34 (15.3%). Marital status was also associated with quitting intention (*χ*^2^ = 14.68, *p* = 0.001); previously married individuals constituted a greater share of those not intending to quit (26.9%) than those who wanted to quit (20.3%). Educational attainment showed a significant relationship with quit intention (*χ*^2^ = 22.93, *p* < 0.001), with smokers lacking formal education more frequently represented among non-intenders (30.1%) than among intenders (22.1%). Previous quit attempts were strongly associated with current intention (*χ*^2^ = 302.40, *p* < 0.001): 38.1% of those not intending to quit had never tried to stop, compared to only 11.0% among those who intended to quit; conversely, 51.4% of intenders had attempted quitting three or more times, versus just 26.6% of non-intenders. Smoking intensity also differed by intention (*χ*^2^ = 18.63, *p* < 0.001), with heavy smoking (20 + cigarettes/day) more common among non-intenders (24.9%) than intenders (19.9%). E-cigarette use was significantly associated with intention to quit (*χ*^2^ = 82.46, *p* < 0.001); over half of non-intenders (53.3%) had never used e-cigarettes, while current use was more common among intenders (18.8% vs. 9.5%). Smoking inside the home was reported more often by non-intenders (63.9% vs. 58.8%, *χ*^2^ = 6.47, *p* = 0.011), as was non-drinking status (23.8% vs. 19.2%, *χ*^2^ = 7.62, *p* = 0.022). Finally, individuals who had not received a doctor’s advice to quit were more likely to lack quit intentions (47.6% vs. 41.2%, *χ*^2^ = 9.79, *p* = 0.002).

**Table 1:** Characteristics of current smokers by intention to quit smoking

Characteristic	Wants to quit (*n* = 1,747)	Does not want to quit (*n* = 904)	Total (*n* = 2,651)	*χ*^2^, *p*-value
Age group, *n* (%)				*χ*^2^ = 98.55, *p* = 0.001
16–24	110 (6.3)	65 (7.1)	175 (6.6)	
25–34	305 (17.5)	138 (15.3)	443 (16.7)	
35–44	344 (19.7)	119 (13.2)	463 (17.5)	
45–54	408 (23.4)	158 (17.5)	566 (21.4)	
55–64	351 (20.1)	172 (19.0)	523 (19.7)	
65 +	229 (13.1)	252 (27.9)	481 (18.2)	
Sex, *n* (%)				*χ*^2^ = 0.77, *p* = 0.381
Male	792 (45.3)	426 (47.1)	1218 (45.9)	
Female	955 (54.7)	478 (52.9)	1433 (54.1)	
Deprivation, *n* (%)				*χ*^2^ = 8.89, *p* = 0.064
Most deprived	539 (30.9)	294 (32.5)	833 (31.4)	
2	439 (25.1)	225 (24.9)	664 (25.1)	
3	317 (18.2)	192 (21.2)	509 (19.2)	
4	272 (15.6)	111 (12.3)	383 (14.4)	
Least deprived	180 (10.3)	82 (9.1)	262 (9.9)	
Marital status, *n* (%)				*χ*^2^ = 14.68, *p* = 0.001
Married/cohabiting	858 (49.1)	407 (45.0)	1265 (47.7)	
Single	534 (30.6)	254 (28.1)	788 (29.7)	
Previously married	355 (20.3)	243 (26.9)	598 (22.6)	
Educational level, *n* (%)				*χ*^2^ = 22.93, *p* < 0.001
Higher education (degree and above)	313 (18.0)	134 (14.9)	447 (16.9)	
Secondary/College	1043 (59.9)	493 (54.7)	1536 (58.1)	
No education	385 (22.1)	275 (30.1)	660 (25.0)	
Quit attempts, *n* (%)				*χ*^2^ = 302.40, *p* < 0.001
Never tried to quit	193 (11.0)	345 (38.1)	538 (20.3)	
Once or twice	656 (37.6)	319 (35.3)	975 (36.8)	
Three times or more	898 (51.4)	240 (26.6)	1138 (42.9)	
Smoking intensity, *n* (%)				*χ*^2^ = 18.63, *p* < 0.001
Light (< 10 cigarettes/day)	616 (35.3)	295 (32.6)	911 (34.4)	
Moderate (10–19 cigarettes/day)	738 (42.2)	342 (37.8)	1080 (40.7)	
Heavy (20 + cigarettes/day)	348 (19.9)	225 (24.9)	573 (21.6)	
Do not know amount smoked	45 (2.6)	42 (4.7)	87 (3.3)	
E-cigarette use status, *n* (%)				*χ*^2^ = 82.46, *p* < 0.001
Current user	329 (18.8)	86 (9.5)	415 (15.7)	
Former user	784 (44.9)	336 (37.2)	1120 (42.3)	
Never user	634 (36.3)	482 (53.3)	1116 (42.0)	
Anyone smokes inside the home, *n* (%)				*χ*^2^ = 6.47, *p* = 0.011
Yes	1028 (58.8)	578 (63.9)	1606 (60.6)	
No	719 (41.2)	326 (36.1)	1045 (39.4)	
Alcohol consumption in the last 12 months, *n* (%)				*χ*^2^ = 7.62, *p* = 0.022
Frequent drinker	856 (49.1)	415 (45.9)	1271 (48.0)	
Occasional drinker	553 (31.7)	274 (30.3)	827 (31.2)	
Non-drinker	335 (19.2)	215 (23.8)	550 (20.8)	
Age started smoking, *n* (%)				*χ*^2^ = 1.95, *p* = 0.163
Started at 16 years and above	960 (55.0)	471 (52.1)	1431 (53.9)	
Started before 16 years	787 (45.0)	433 (47.9)	1220 (46.1)	
Self-rated health, *n* (%)				*χ*^2^ = 1.34, *p* = 0.512
Very good/good	980 (56.1)	488 (54.0)	1468 (55.4)	
Fair	440 (25.2)	245 (27.1)	685 (25.8)	
Bad/very bad	327 (18.7)	171 (18.9)	498 (18.8)	
Has long-standing illness, *n* (%)				*χ*^2^ = 1.34, *p* = 0.248
Yes	993 (56.8)	535 (59.2)	1528 (57.6)	
No	754 (43.2)	369 (40.8)	1123 (42.4)	
Doctor advised to quit smoking, *n* (%)				*χ*^2^ = 9.79, *p* = 0.002
Yes	1027 (58.8)	474 (52.4)	1501 (56.6)	
No	720 (41.2)	430 (47.6)	1150 (43.4)	

Values are unweighted counts and percentages. *χ*^2^ tests were calculated using unweighted data for descriptive comparison

**Fig. 1:**
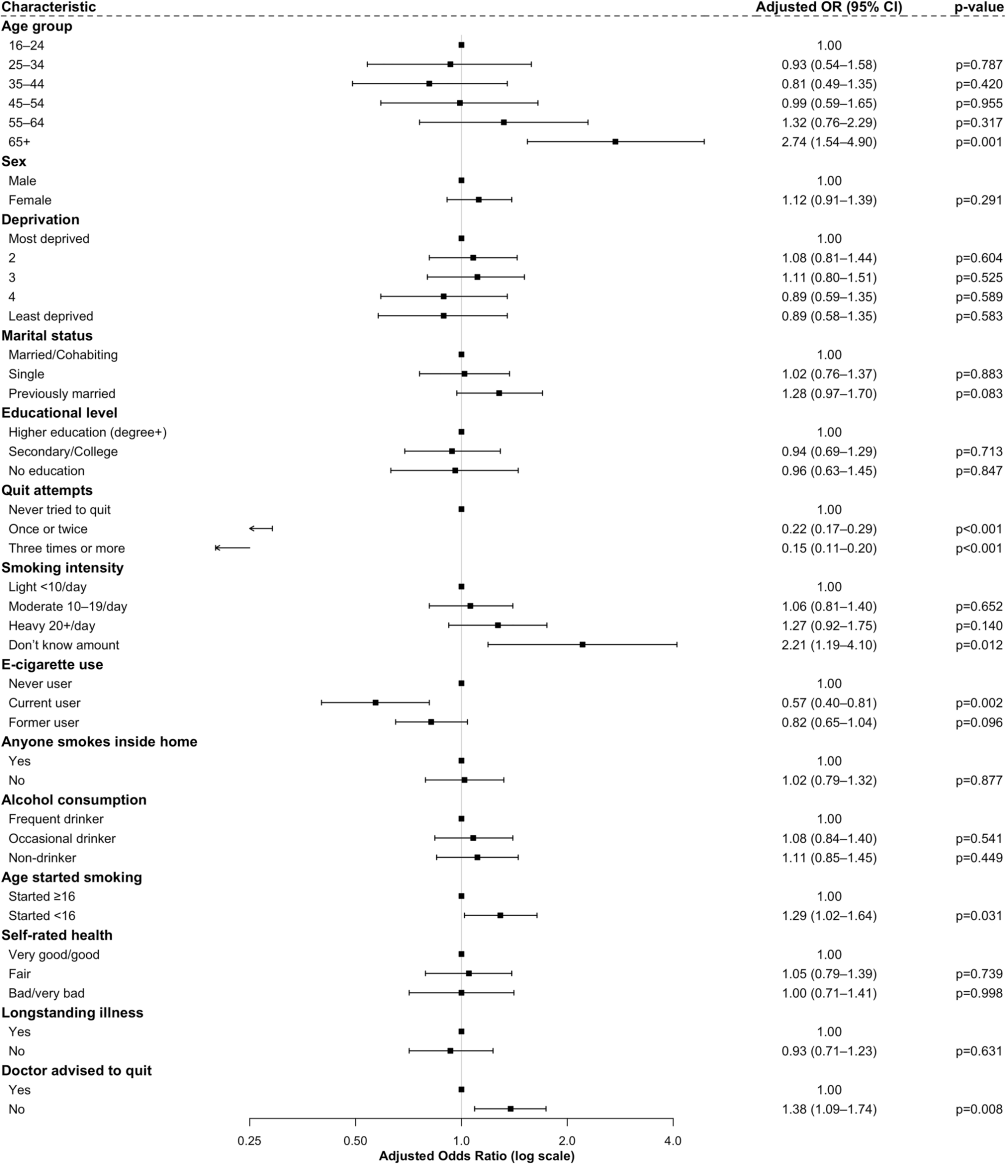
Adjusted odds ratios (95% CI) for no intention to quit smoking. Odds ratios are from a single multivariable model including all covariates; reference categories are shown at odds ratio = 1.00

Table 2 displays the crude and adjusted odds ratios (ORs) for the association between various characteristics and no intention to quit smoking among current smokers. In adjusted analyses, older age was significantly associated with no intention to quit; individuals aged 65 years and above had over twice the odds of not intending to quit compared to those aged 16–24 years (adjusted OR [aOR] = 2.74, 95% CI 1.54–4.90, *p* = 0.001). Having started smoking before the age of 16 was also associated with higher odds of no quit intention (aOR = 1.29, 95% CI 1.02–1.64, *p* = 0.031). A strong inverse association was observed with previous quit attempts: compared to those who had never tried to quit, individuals who had tried once or twice (aOR = 0.22, 95% CI 0.17–0.29, *p* < 0.001) or three or more times (aOR = 0.15, 95% CI 0.11–0.20, *p* < 0.001) were significantly less likely to report no intention to quit. Current e-cigarette users were also less likely to lack quit intentions compared to never users (aOR = 0.57, 95% CI 0.40–0.81, *p* = 0.002), whereas former users did not differ significantly. Individuals who did not know how many cigarettes they smoked per day had higher odds of no intention to quit (aOR = 2.21, 95% CI 1.19–4.10, *p* = 0.012). Additionally, those who had not received a doctor’s advice to quit were more likely to lack intention (aOR = 1.38, 95% CI 1.09–1.74, *p* = 0.008). Other variables, including sex, deprivation, marital status, education, anyone smokes inside home, alcohol consumption, self-rated health, and long-standing illness, were not significantly associated with quitting intentions in the fully adjusted model (See Fig. 1).

**Table 2:** Crude and adjusted odds ratios for no intention to quit smoking among current smokers

Characteristic	Crude OR (95% CI), *p*-value	Adjusted OR (95% CI), *p*-value
Age group		
16–24	Reference	Reference
25–34	0.77 (0.53–1.10), *p* = 0.154	0.93 (0.54–1.58), *p* = 0.787
35–44	0.59 (0.40–0.85), *p* = 0.005	0.81 (0.49–1.35), *p* = 0.420
45–54	0.66 (0.46–0.94), *p* = 0.020	0.99 (0.59–1.65), *p* = 0.955
55–64	0.83 (0.58–1.18), *p* = 0.304	1.32 (0.76–2.29), *p* = 0.317
65 +	1.86 (1.31–2.66), *p* = 0.001	2.74 (1.54–4.90), *p* = 0.001
Sex		
Male	Reference	Reference
Female	0.93 (0.79–1.09), *p* = 0.381	1.12 (0.91–1.39), *p* = 0.291
Deprivation		
Most deprived	Reference	Reference
2	0.93 (0.76–1.16), *p* = 0.569	1.08 (0.81–1.44), *p* = 0.604
3	1.11 (0.88–1.40), *p* = 0.370	1.11 (0.80–1.51), *p* = 0.525
4	0.75 (0.58–0.97), *p* = 0.030	0.89 (0.59–1.35), *p* = 0.589
Least deprived	0.84 (0.62–1.12), *p* = 0.235	0.89 (0.58–1.35), *p* = 0.583
Marital status		
Married/cohabiting	Reference	Reference
Single	1.00 (0.83–1.21), *p* = 0.978	1.02 (0.76–1.37), *p* = 0.883
Previously married	1.44 (1.18–1.76), *p* < 0.001	1.28 (0.97–1.70), *p* = 0.083
Educational level		
Higher education (degree and above)	Reference	Reference
Secondary/college	1.10 (0.88–1.39), *p* = 0.397	0.94 (0.69–1.29), *p* = 0.713
No education	1.67 (1.29–2.15), *p* < 0.001	0.96 (0.63–1.45), *p* = 0.847
Quit attempts		
Never tried to quit	Reference	Reference
Once or twice	0.27 (0.22–0.34), *p* < 0.001	0.22 (0.17–0.29), *p* < 0.001
Three times or more	0.15 (0.12–0.19), *p* < 0.001	0.15 (0.11–0.20), *p* < 0.001
Smoking intensity		
Light (< 10 cigarettes/day)	Reference	Reference
Moderate (10–19 cigarettes/day)	0.97 (0.80–1.17), *p* = 0.733	1.06 (0.81–1.40), *p* = 0.652
Heavy (20 + cigarettes/day)	1.35 (1.09–1.68), *p* = 0.007	1.27 (0.92–1.75), *p* = 0.140
Do not know amount smoked	1.95 (1.25–3.03), *p* = 0.003	2.21 (1.19–4.10), *p* = 0.012
E-cigarette use status		
Current user	0.56 (0.47–0.67), *p* < 0.001	0.57 (0.40–0.81), *p* = 0.002
Former user	0.34 (0.26–0.45), *p* < 0.001	0.82 (0.65–1.04), *p* = 0.096
Never user	Reference	Reference
Anyone smokes inside the home		
Yes	Reference	Reference
No	0.81 (0.68–0.95), *p* = 0.011	1.02 (0.79–1.32), *p* = 0.877
Alcohol consumption in the last 12 months		
Frequent drinker	Reference	Reference
Occasional drinker	1.02 (0.85–1.23), *p* = 0.819	1.08 (0.84–1.40), *p* = 0.541
Non-drinker	1.32 (1.08–1.63), *p* = 0.008	1.11 (0.85–1.45), *p* = 0.449
Age started smoking		
Started at 16 years and above	Reference	Reference
Started before 16 years	1.12 (0.95–1.32), *p* = 0.163	1.29 (1.02–1.64), *p* = 0.031
Self-rated health		
Very good/good	Reference	Reference
Fair	1.12 (0.92–1.35), *p* = 0.250	1.05 (0.79–1.39), *p* = 0.739
Bad/very bad	1.05 (0.85–1.30), *p* = 0.655	1.00 (0.71–1.41), *p* = 0.998
Has long-standing illness		
Yes	Reference	Reference
No	0.91 (0.77–1.07), *p* = 0.248	0.93 (0.71–1.23), *p* = 0.631
Doctor advised to quit smoking		
Yes	Reference	Reference
No	1.29 (1.10–1.52), *p* = 0.002	1.38 (1.09–1.74), *p* = 0.008

Odds ratios are from a single multivariable model including all covariates

Model diagnostics indicated that the assumptions of logistic regression were adequately met. Variance inflation factors showed no evidence of problematic multicollinearity (all VIFs < 5; mean VIF = 2.27; reference: VIF < 10 generally indicates acceptable levels, with < 5 preferred). The survey-adjusted goodness-of-fit test, Archer–Lemeshow F-adjusted mean residual test, suggested that the final model fit the data well (*F* = 0.71, *p* = 0.70; *p* > 0.05 indicates no lack of fit). The Brier score was 0.187 (95% CI 0.180–0.195), indicating acceptable predictive accuracy (reference: values closer to 0 show perfect accuracy, with < 0.25 typically considered acceptable for binary outcomes).

**Fig. 2:**
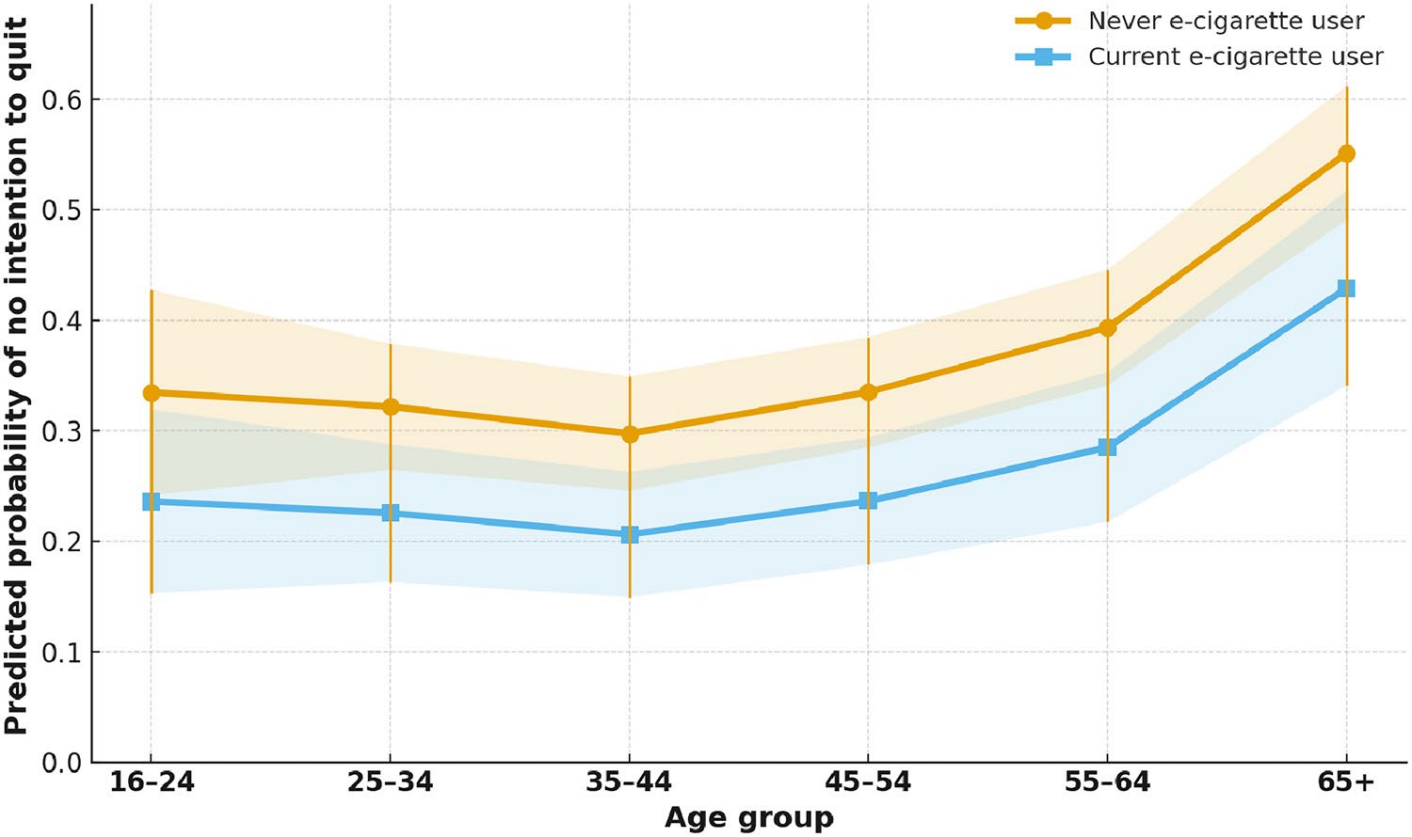
Predicted probability of reporting no intention to quit smoking by age group and e-cigarette use. Predicted probabilities were derived from a survey-weighted logistic regression model adjusting for sex, socioeconomic deprivation, marital status, education, smoking intensity, previous quit attempts, anyone smoking in the home, age of smoking initiation, alcohol use, self-rated general health, doctor’s advice to quit, and long-standing illness. The probability of no quit intention increased with age, with consistently lower probabilities among current e-cigarette users compared with never users. Although the pattern was consistent, the age-by-e-cigarette interaction was not statistically significant

Fig. 2 presents the adjusted predicted probabilities of reporting no intention to quit smoking by age group and e-cigarette use status. Among never e-cigarette users, the predicted probability of having no quit intention was 33% (95% CI 24–43) in the 16–24 age group, 32% (95% CI 26–38) at ages 25–34, 30% (95% CI 25–35) at ages 35–44, 33% (95% CI 29–38) at ages 45–54, 39% (95% CI 34–45) at ages 55–64, and 55% (95% CI 49–61) among those aged 65 and older. Current e-cigarette users had consistently lower probabilities across all age groups: 24% (95% CI 15–32) for ages 16–24, 23% (95% CI 16–29) for ages 25–34, 21% (95% CI 15–26) for ages 35–44, 24% (95% CI 18–29) for ages 45–54, 29% (95% CI 22–35) for ages 55–64, and 43% (95% CI 34–52) for ages 65 +. The gap between never and current users was evident across all age groups, although the age-by-e-cigarette interaction was not statistically significant (*p* for interaction > 0.05).

As an additional sensitivity analysis, the survey year was included in the final models. This adjustment did not result in any statistically significant changes in the odds ratios, suggesting that the observed associations were consistent across survey years and not influenced by temporal effects. The results presented are based on models that exclude the year adjustment, as it did not meaningfully improve model fit or alter the findings.

## Discussion

This study provides important insights into the factors associated with having no intention to quit smoking among individuals in Scotland, contributing to the growing body of research on hard-to-reach smoker populations. The finding that older adults (65 + years) were significantly more likely to report no quit intention aligns with previous studies showing that long-term smokers often develop a strong behavioural and psychological dependence on tobacco, which may make cessation appear less attainable or less relevant later in life [15, 16]. This is particularly concerning given Scotland's aging population and the compounding health risks that smoking poses for older adults, who are more vulnerable to chronic diseases. It is also important to note that lower quit intention among older smokers may reflect not only behavioural dependence but also the long-standing neglect of this group in tobacco control policy and research, suggesting the need for interventions tailored specifically to their circumstances. Our findings also raise the possibility that harm reduction approaches, such as providing access to and information about reduced-risk nicotine products, including e-cigarettes, could be explored as one pragmatic option for reducing health harms in a group otherwise showing limited motivation to quit. Similarly, the association between early smoking initiation and lower quit intention suggests that early nicotine exposure may be linked to more entrenched patterns of addiction, potentially due to effects on the developing brain. However, early initiation may also reflect broader vulnerability to smoking, including social and potentially genetic predispositions [15], underlining that this association should not be interpreted as purely causal. These findings highlight the need for targeted interventions that address the unique barriers faced by these subgroups, such as age-specific cessation messaging or programmes that emphasise the health benefits of quitting at any age [17, 18].

Our adjusted predictive probability estimates further illustrate these patterns. Across all age groups, current e-cigarette users consistently had a lower probability of reporting no intention to quit compared with never users, even after adjustment for sociodemographic, behavioural, and health-related covariates. For example, among adults aged 65 and older, the adjusted probability of having no quit intention was 42.9% for current e-cigarette users compared with 55.1% for never users. While the age-by-e-cigarette interaction was not statistically significant, the consistent direction of the probabilities suggests that e-cigarette use may be associated with lower quit resistance across the life course. These findings raise the possibility that age-tailored harm reduction strategies could help engage smokers with low cessation motivation, particularly older adults, by presenting reduced-risk nicotine products as a potential option for those less inclined to attempt quitting altogether.

The role of healthcare providers emerged as another critical factor, with smokers who had never received a doctor’s advice to quit being significantly more likely to lack intention to stop. This underlines a gap in current clinical practice, where smoking cessation is often not prioritised during routine medical visits, especially for patients who do not express interest in quitting. Given that healthcare encounters represent a key opportunity to influence behaviour change, systematic implementation of brief advice protocols, even for resistant smokers, could help shift attitudes over time. Recent evidence highlights this point: many healthcare providers report being less comfortable delivering cessation interventions to patients who are not ready to quit, highlighting the need for improved training and system-level support [19].

The lack of association between socioeconomic deprivation and quit intention was somewhat surprising, as deprivation is a well-established determinant of smoking prevalence. Because social disadvantage was an initial hypothesis, its absence here deserves explicit attention. This suggests that while deprivation strongly predicts smoking uptake and persistence, it may not directly shape motivation to quit. Instead, deprivation likely operates through structural barriers, such as stress, financial strain, and limited access to cessation resources that affect actual quit success rather than stated intention [3]. Supporting this, a recent French cohort study found that smokers in deprived neighbourhoods had significantly lower quit rates even after accounting for individual socioeconomic status, highlighting the role of contextual disadvantage in shaping cessation outcomes [20]. This distinction is crucial for policymakers, as it suggests that interventions must address both motivation and the external obstacles that prevent motivated smokers from succeeding.

The strong inverse relationship between previous quit attempts and no quit intention reinforces the idea that even unsuccessful attempts are often associated with greater readiness to quit [21]. Still, this association may also reflect continuity of motivation, whereby those who tried to quit in the past are more likely to remain motivated in the present, rather than quit attempts themselves generating intention. Public health messaging could leverage this by normalising relapse as part of the quitting process and encouraging smokers to view each attempt as progress.

Similarly, the finding that current e-cigarette users were less likely to lack quit intention contributes to the ongoing debate about the role of vaping in harm reduction. While our results cannot establish causality, they are consistent with the possibility that reduced-risk nicotine products may play a role in supporting motivation to quit among smokers who are otherwise resistant. Some evidence suggests that even smokers with no initial quit intention who adopt e-cigarettes may eventually transition away from cigarettes altogether [17], and Cochrane reviews indicate that e-cigarettes can be more effective for cessation than nicotine replacement therapy [22, 23]. In contrast, former e-cigarette use was not associated with higher quit intention, which may reflect widespread misperceptions among adults that e-cigarettes are as harmful, or even more harmful, than smoking. Addressing these misperceptions through campaigns clarifying the continuum of risk across nicotine products could help prevent smokers from abandoning e-cigarettes and returning exclusively to cigarettes. The elevated odds of no quit intention among smokers who were unaware of their daily cigarette consumption point to a subgroup that might benefit from interventions to increase self-monitoring, such as smartphone apps or structured smoking diaries [24]. Yet this association, too, should not be interpreted causally, lack of awareness may simply reflect indifference rather than inability, highlighting the importance of distinguishing carefully between capability and motivation in designing interventions.

Finally, the persistence of a substantial proportion of smokers with no quit intention (34.1%) poses a significant challenge to Scotland’s ambitious 2034 tobacco-free goal. Current policies and interventions tend to focus on smokers who are already motivated to quit, leaving a gap in strategies for engaging resistant individuals. To address this, a multi-pronged approach is needed, combining targeted messaging for high-risk subgroups (e.g. older adults, early initiators, marginalised populations), systemic changes in healthcare delivery (e.g. universal cessation advice), and a stronger emphasis on harm reduction approaches that meet smokers where they are, supporting safer nicotine substitution without requiring immediate abstinence. The findings also suggest the importance of reframing cessation as a gradual process, where small steps, like reducing cigarette consumption or exploring alternatives, are validated as progress. This nuanced approach could help shift social norms around smoking and create an environment where quitting feels more achievable, even for those who initially resist. Emerging strategies such as culturally tailored interventions have been shown to improve quit outcomes compared to generic approaches [25], while educational campaigns can also increase cessation motivation in resistant groups [26–28]. Together, such approaches suggest a need for interventions that are context sensitive, culturally relevant, and designed to engage smokers who may not initially intend to quit.

The findings of this study have several important implications for tobacco control policies and clinical practice in Scotland and beyond. First, the strong association between healthcare provider advice and quit intention underlines the need to integrate systematic, non-judgemental smoking cessation interventions into all healthcare settings. This could include training providers to deliver brief advice consistently, implementing electronic prompts in patient records, and offering tailored support for resistant smokers, such as motivational interviewing techniques. Second, the identification of high-risk subgroups, such as older adults, early initiators, and those unaware of their smoking intensity, calls for targeted public health campaigns that address the specific barriers and misconceptions these groups face. For example, messaging for older smokers could emphasise the immediate benefits of quitting, such as improved circulation and reduced shortness of breath, rather than long-term gains [29]. Third, the potential role of e-cigarettes in reducing quit resistance suggests that harm reduction strategies should be carefully incorporated into tobacco control policies, with clear guidelines to prevent unintended uptake among non-smokers [30–32]. Finally, the study highlights the need for community-based interventions that address the social and environmental determinants of smoking, particularly in deprived areas where smoking rates remain high. This could involve partnerships with local organisations to create smoke-free spaces, peer support programmes, and initiatives that address the broader determinants of health, such as poverty and mental health. Evidence from France demonstrates that structural disadvantage undermines cessation despite individual motivation [20], while systematic reviews confirm the effectiveness of culturally tailored approaches in diverse settings [25]. By adopting a comprehensive, multi-level approach, policymakers and practitioners can better support all smokers, including those who are resistant to quitting, and move closer to Scotland’s 2034 tobacco-free vision.

This study has several notable strengths, including its large, nationally representative sample and the use of robust statistical methods to control for potential confounders. The inclusion of a wide range of sociodemographic, behavioural, and health-related variables provides a comprehensive picture of the factors associated with quit intention, offering valuable insights for both research and practice. However, the study also has limitations that must be considered. The cross-sectional design precludes causal inferences, meaning we cannot determine whether the identified factors directly influence quit intention or are merely associated with it. Additionally, the reliance on self-reported data may introduce recall or social desirability bias, particularly for sensitive behaviours like smoking. Future research should employ longitudinal designs to explore causal pathways and incorporate qualitative methods to better understand the lived experiences of smokers with no quit intention. Despite these limitations, the study provides critical evidence to inform targeted interventions and policies aimed at reducing smoking prevalence in Scotland.

## Conclusion

This research provides important insights into the characteristics of smokers in Scotland who report no intention to quit, a group that remains largely overlooked in tobacco control efforts. The findings reveal that older age, early smoking initiation, lack of healthcare engagement, and unawareness of smoking intensity are significantly associated with a lack of motivation to stop smoking. In contrast, previous quit attempts and current e-cigarette use are strong indicators of quit intention, even if cessation has not yet been achieved. These results highlight the importance of broadening the focus of cessation strategies beyond ready-to-quit populations by incorporating interventions that specifically target unmotivated smokers. By adopting a more inclusive and tailored approach, Scotland can make meaningful progress toward its 2034 tobacco-free goal while addressing the persistent health inequalities associated with smoking.

## Data Availability

To download the dataset used in the analyses, please visit https://ukdataservice.ac.uk/finddata/browse/health/

## Declarations

### Research involving human and/or animals participants

As this study involved secondary data analysis, no ethical approval or informed consent was required by the authors for this research, as it qualifies for exemption from human subject research.
